# Global correlates of range contractions and expansions in terrestrial mammals

**DOI:** 10.1038/s41467-020-16684-w

**Published:** 2020-06-05

**Authors:** Michela Pacifici, Carlo Rondinini, Jonathan R. Rhodes, Andrew A. Burbidge, Andrea Cristiano, James E. M. Watson, John C. Z. Woinarski, Moreno Di Marco

**Affiliations:** 1 Global Mammal Assessment programme, Dipartimento di Biologia e Biotecnologie “Charles Darwin”, Sapienza Università di Roma, Viale dell’Università 32, I-00185 Rome, Italy; 2 School of Earth and Environmental Sciences, The University of Queensland, Brisbane, QLD 4072 Australia; 3 87 Rosedale Street, Floreat, WA 6014 Australia; 4 Wildlife Conservation Society, Global Conservation Program, Bronx, New York, NY USA; 5 Threatened Species Recovery Hub of the National Environment Science Program, Research Institute for the Environment and Livelihoods, Charles Darwin University, Darwin, NT 0909 Australia; 6 Dipartimento di Biologia e Biotecnologie “Charles Darwin”, Sapienza Università di Roma, Rome, I-00185 Italy; 7 CSIRO Land and Water, EcoSciences Precinct, 4102 Brisbane, Australia

**Keywords:** Climate-change ecology, Conservation biology

## Abstract

Understanding changes in species distributions is essential to disentangle the mechanisms that drive their responses to anthropogenic habitat modification. Here we analyse the past (1970s) and current (2017) distribution of 204 species of terrestrial non-volant mammals to identify drivers of recent contraction and expansion in their range. We find 106 species lost part of their past range, and 40 of them declined by >50%. The key correlates of this contraction are large body mass, increase in air temperature, loss of natural land, and high human population density. At the same time, 44 species have some expansion in their range, which correlates with small body size, generalist diet, and high reproductive rates. Our findings clearly show that human activity and life history interact to influence range changes in mammals. While the former plays a major role in determining contraction in species’ distribution, the latter is important for both contraction and expansion.

## Abstract (web summary)

Understanding why many species ranges are contracting while others are stable or expanding is important to inform conservation in an increasingly human-modified world. Here, Pacifici and colleagues investigate changes in the ranges of 204 mammals, showing that human factors mostly explain range contractions while life history explains both contraction and expansion.

## Introduction

The impact of human activities on the environment is resulting in the alteration of major macroecological patterns that characterise life on Earth, including the loss of top predators^1^ and other apex consumers^2^, and the reduction of average body size in animal communities^3^. Since the 1970s, the human population has doubled, leading to increased anthropogenic pressures^4^ that have caused the size of animal populations to fall by almost half^5^. Mammals, in particular, have experienced serious declines, with 1210 (25%) species now considered threatened with extinction^6^. Declines in this period have been documented for ungulates and carnivores, where for every species that improved its Red List status, eight have deteriorated^7^. These trends likely started before the beginning of the third industrial revolution, in combination with shifting cultural values that led to the overexploitation of natural resources and an increase in land pollution^8^. Broad-scale destruction of natural habitats, overexploitation of natural resources and competition with/predation by invasive alien species are the primary source of terrestrial biodiversity loss^9,10^. However, the relationship between human activities and species distribution and abundance is complex^11^, and there are several examples where the same activity can have either positive or negative effects, depending on the species and the geographic area considered^12^. For example, the expansion of oil palm plantations in Sumatra led to a steep decline in the populations of Sumatran tiger (*Panthera tigris sumatrae*), but benefitted populations of adaptable and invasive species like the wild boar (*Sus scrofa*), a species that has undergone a 100-fold increase in population density in the last 24 years^13^. In North America, oil sand development in boreal forest has led to precipitous declines in moose (*Alces alces*), but marked increases in white-tailed deer (*Odocoileus virginianus*) populations^14^.

Understanding the mechanisms underlying recent dynamics in species distribution ranges is essential to predict future human influences on biodiversity, and guide conservation responses. Range dynamics vary across taxa and space due to the combined effects of environmental factors, biotic interactions, human impact and species’ life histories^15,16^. Several studies on different taxonomic groups have attempted to identify the relative importance of life-history traits in determining range size and change^17,18^. Identifying common life-history traits of species that have shown range declines and expansions can help to provide a basis for predicting which species are more sensitive to extinction^18^, but also those that will likely expand and colonise new areas^19^. However, these traits alone usually fail to explain the complexity of range dynamics due to the difficulty of coupling these data with extrinsic factors for a large number of species^15^. In addition, previous studies on range dynamics have only considered local or regional changes in species’ distribution, lacking a global view of the impacts of global change on the rate of range change (e.g. see ref. ^20^ for North American carnivores and ungulates).

Here, we compare the past (1970s) and current (2017) distributions of terrestrial non-volant mammals to evaluate the extent of recent range contractions and expansions. We focus our analyses on mammals because they have greater availability of past distribution data, and show much larger range contractions compared with other taxonomic groups^21–23^. We use the Pacifici et al.^24^ database on past range maps for 204 species of mammals, broadly representative of major terrestrial biomes and taxonomic groups, and then compare the 1970s maps with the current distribution ranges of species from the International Union for Conservation of Nature^6^ (IUCN) to calculate the extent of range contractions and/or expansions for each species. We looked at human pressures, in particular land-use and climate change, and intrinsic characteristics of species as potential drivers of distribution change^16,25,26^. We quantify the relative contributions of these variables in explaining the observed distribution changes. We perform our analysis at a spatial resolution of 100 km^2^, and a sensitivity test at 10,000 km^2^ for comparison.

We find that the vast majority of species lost part of their past range over the five-decade period, while just one in five species expanded their past range to some extent. Anthropogenic factors play a major role in determining contraction in species’ ranges and a smaller role in determining expansion, while life-history traits are important for both range contraction and expansion. Our findings provide clear evidence of how human activity and life history interact to influence range changes in mammals, thus suggesting that management solutions aimed at preventing species’ extinction should take into account both intrinsic characteristics of animals and external threats.

## Results

### Changes in the sizes of species’ ranges

**Fig. 1:**
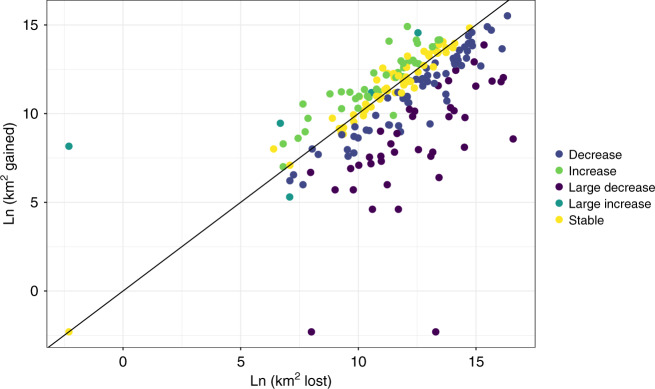
Scatterplot showing the relationship between increase and decrease in the range of each species. Classes refer to the percentage range change with respect to the 1970s: large decrease (>50%), moderate decrease (between 49 and 5%), stable (within 5%), moderate increase (between 5 and 49%) and large increase (>50%).

We found that 40 species (20%) show a large decrease (>50%) in their distribution range since the 1970s, and 65 (32%) moderate decreases (between 5 and 49%, Fig. 1). When considering the opposite trend, 4 species (2%) show large increases in their range (>50%) and 39 species (19%) moderate increases (between 5 and 49%, Fig. 1). Finally, 57 species (28%) remained relatively stable (positive and negative changes were below the 5% thresholds). Stable species generally show a similar small amount of range lost and gained, indicating slight shifts in the distribution area rather than no changes at all (Fig. 1, Supplementary Fig. 2).

### Predictors of range contraction

**Fig. 2:**
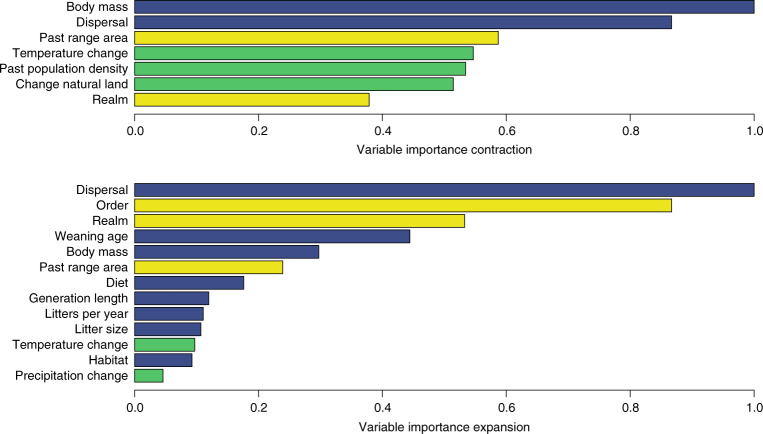
Random forest variable importance plots for selected predictors in the range contraction and expansion models. The plot shows the decreasing importance of intrinsic and extrinsic variables at 10-km resolution in predicting proportional loss (upper panel) and gains (lower panel) in species’ ranges. Big changes in these measures indicate important variables. Yellow bars indicate life-history traits, blue bars human-pressure variables and red bars other variables. *n* = 204 species have been analysed.

**Fig. 3:**
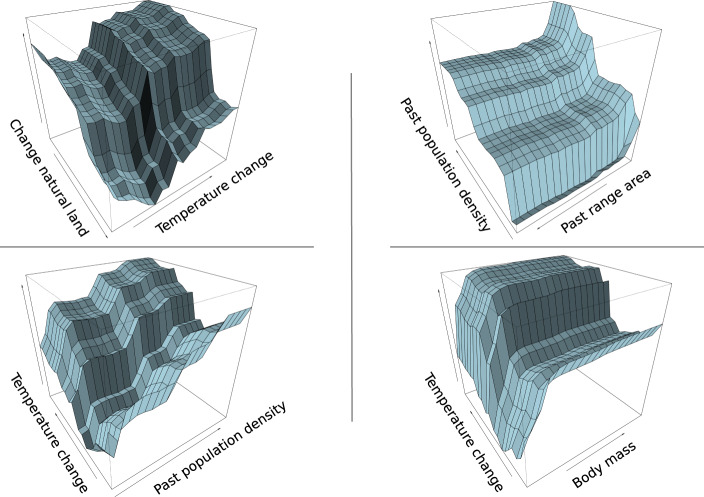
Partial dependence plots to show the effects of selected pairs of predictors on range contraction. Top-left plot shows the negative interaction of change in natural lands with mean annual temperature; the top-right plot represents the negative interaction of human population density in the past with the size of the past range on range contraction; the bottom-left plot describes the positive interaction of change in mean annual temperature with past human population density; the bottom-right plot illustrates the positive interaction of body mass with mean annual temperature change on range contraction.

We define range contraction as the disappearance of a species from part of its past range. Overall, both life-history and extrinsic variables play an important role in determining such contraction, but human pressures have a prominent role, when measuring variable importance using a Random Forest model for regression^27^ (Fig. 2). Species with large body mass, in particular, experienced large (up to >70%) range contractions, as well as those species with very low and very high dispersal rates (Supplementary Fig. 3). The most important extrinsic drivers of contraction are high human population density in the past, the increase in mean annual temperature and the loss of natural land use (Figs. 2 and 3). This result is confirmed when employing a different analytical resolution (100 km instead of 10 km) to quantify anthropogenic variables within species’ ranges (Supplementary Fig. 4). The interaction between the increase in temperature and the intensification of human activities has a magnified effect on range contraction, and species with restricted distribution are more susceptible to lose parts of their range, especially if these were densely populated by humans (Fig. 3).

Australasian species have the highest percentage of range loss, followed by Palaearctic and Indo-Malayan species (Supplementary Fig. 3). Overall, the best model obtained by using the MIR metric is able to explain 34.53% of the variance in observed range contraction (Supplementary Fig. 7).

### Predictors of range expansion

**Fig. 4:**
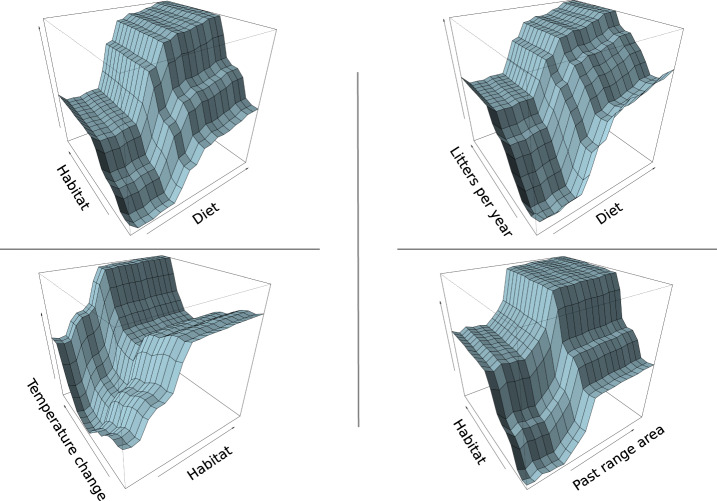
Partial dependence plots to show the effects of selected pairs of predictors on range expansion. Top-left plot represents the positive interaction of dietary breadth with habitat breadth; the top-right plot shows the positive interaction of dietary breadth with litters per year; the bottom-left plot describes the positive interaction of change in mean annual temperature with habitat breadth; the bottom-right plot illustrates the positive interaction of habitat breadth with the size of the past range on range expansion.

We define range expansion as the colonisation of areas outside the past range of a species, within its total potential dispersal reach throughout the study period. Life-history traits and biogeography are the most important predictors in the range expansion model (Figs. 2 and 4); this result is also supported by a sensitivity analysis where the dispersal reach is estimated in a more conservative way (Supplementary Fig. 5). Dispersal and taxonomic order are the most important predictors, with orders characterised by small-bodied species showing higher proportional gains (i.e. *Afrosoricida*, *Cingulata*, *Didelphimorphia*, *Macroscelidea*, *Microbiotheria*, *Peramelemorphia* and *Rodentia*). This trend is also evident from the partial dependence plot of body mass, where the curve approximated a rectangular hyperbole (Supplementary Fig. 6). Notably, this trend is opposite to that observed for the same variable in the contraction model. An increase in range size is positively related to the number of litters produced per year and to dietary breadth (and their interaction, Fig. 4). The relationship with weaning age is less clear, with an initial steep decrease in the proportion of range gained when weaning age increases (up to 100 days), and an inversion of this trend afterwards (Supplementary Fig. 6). It is interesting to note that species with high reproductive rates that are generalists in terms of diet and/or habitat have expanded their ranges the most (Fig. 4).

Species living in the Americas have the highest percentages of range increase compared wih those living in the other biogeographic realms. Increases in temperature are positively associated with expansion (Supplementary Fig. 6). Among non-climatic drivers, the most meaningful external pressure related to human activities is the proportion of buildings within a given cell, where the lower the increase, the higher the percentage of range gained by the mammal species considered (Supplementary Fig. 6). Overall, the best model obtained with the variable selection procedure is able to explain 32.62% of the variance in observed range expansion within the buffer (Supplementary Fig. 8).

## Discussion

Understanding contemporary range contractions and expansions is essential to disentangle the mechanisms that drive species’ responses to habitat modification by humans. Anthropogenic and life-history factors have significantly influenced the distribution of mammals in the recent past. We found that while both range contractions and expansions have occurred, the number of mammal species that underwent an overall decrease in range size since the 1970s was twice the number of species that underwent an overall increase. In agreement with previous findings^28^, our results also show that the distribution of species has changed in the last 5 decades, with marked differences among geographic regions and taxonomic orders. Life-history traits were associated with both range contractions and expansions, while human activities were key in predicting contractions, but had lower importance as predictors of expansion. This indicates that species that are intrinsically able to rapidly colonise new areas can do so almost regardless of human pressures, and are less likely to undergo an overall decrease in range size. Instead, species that are intrinsically unable to cope with high human pressure are likely to lose part of their range if human activities intensify, but are unlikely to expand towards new areas at a sufficiently fast pace to prevent overall reductions in range size.

Some variables had high predictive importance in both our contraction and expansion models. Body mass, for example, was one of the most important factors affecting range changes in both directions. We found that many large-mammal species lost large portions of their past range, and few managed to expand. Large-bodied species, especially carnivores, are usually characterised by higher dispersal abilities since locomotion becomes energetically cheaper as body mass increases, allowing larger daily movements^29,30^. In addition, all else being equal, species with larger body sizes need larger home ranges, and have smaller populations, so they may not be able to cope well with habitat fragmentation and loss^31^. Theoretically, large-bodied non-volant mammals should be able to rapidly move to more suitable areas due to their long dispersal capacity^32^. However, direct human pressure remains the highest threat to these species, and likely prevents their populations from expanding and establishing in new sites^33^. In contrast, small-bodied species had the highest proportion of range expansions. These are mostly represented by r-selected species in our sample, suggesting that they are those with the best chance to rapidly adapt to new areas.

The size of the past geographic range was an important predictor in both models, where species with the largest 1970s distributions are associated with lower percentage declines and higher percentage increases. Species with small ranges may be more subject to localised pressures and stochastic events^34^, and their small range may be determined to some extent by habitat specialisation. In contrast, large-range species are more likely exposed to a higher variety of habitats, and show higher adaptability to different environments^35^. This hypothesis is further supported by habitat specificity, being an important predictor of both range contraction and expansion (with opposite trends).

Biogeography was another key parameter in determining range dynamics. Species living in Australasia experienced the most dramatic range declines in the last few decades, confirming previous analyses^36^. Much of the recent loss of Australian terrestrial mammals has occurred in areas with low human population pressures, and where native vegetation has not been significantly removed, particularly in the interior deserts and tropical savannas^37^. Contrary to what has happened in other continents, where the main causes of extinction were habitat loss and overhunting^10^, the decline of most Australian species is directly related to predation by introduced species, particularly the European red fox, *Vulpes vulpes* and the feral cat, *Felis catus*, and changes in fire regimes^37^. Therefore, as expected, controlling for biogeographic realm was important to account for the different pressures operating in different regions. Contrasting pressures and impacts (i.e. high disturbance rates) occurred in the Indo-Malayan realm, the second most affected realm in terms of species’ range contraction according to our findings, where annual deforestation rate is the highest recorded in the tropics since 1990^38^. In these areas, human population density has steadily increased at a rate of >1.5% per annum over the past five decades (https://unstats.un.org/home/), and the percentage of terrestrial non-volant mammals currently threatened with extinction is 30% (compared with the global 25% estimate^6^). Species living in the Americas showed the highest increases in range size. In our sample, the majority of them are small-bodied, generalist species (74%), probably able to exploit a great number of habitats and resources, including modified ones. In agreement with previous findings^20^, large mammals (carnivores and especially ungulates) and primates experienced important range declines also in these areas, and we show that human pressure and climatic change interacted with life-history traits in determining these declines.

Among anthropic drivers, the past density of human population and the steep decrease in natural land were the most important predictors of range contraction. One of the main issues associated with urbanisation is that animals are increasingly exposed to urban boundaries with different edge contrasts—different composition between adjoining ecosystems^39^—and boundaries with high contrast likely prevent the movement of animals across landscapes^40,41^. This concept is related to fragmentation, and can influence the distribution of species at local scales^40^. This result is in agreement with previous findings on the effects of fragmentation on terrestrial mammals^42^, showing that the most threatened species are those subjected to the most fragmentation, have small distributions and low proportions of suitable habitat within their ranges. The Indian rhinoceros (*Rhinoceros unicornis*) represents one of the most dramatic examples, having lost >99% of its range in the last five decades due to widespread conversion of alluvial plain grasslands to agricultural areas, habitat degradation and poaching. Its remaining populations are highly fragmented and mostly concentrated in a single protected site, surrounded by areas densely populated by humans, which prevents possible range increase^43^.

In the range expansion model, life-history traits tend to confirm known theories on r-selected and generalist species^44^. In particular, our results support previous studies that found a positive relationship between a reduced interval between reproductive events and the spread rate of mammal species^45^. Fast reproductive rates and low dietary specialisation make species able to exploit unpredictable and stressed environments, thus favouring range expansions^45^. As an example, the Nine-banded Armadillo (*Dasypus novemcinctus*), a species with broad diet and habitat breadth and high reproduction rates^46^, showed the largest percentage range increase in our analysis (73% of the potential expansion buffer occupied).

Despite mammals being one of the most studied animal groups, finding reliable information on their past ranges is challenging, especially for those species largely distributed (e.g. Palaearctic species, which are underrepresented in our sample). Field surveys in the past were largely biased towards sites of particular natural beauty, high species richness and areas near experts’ residences/research centres^47^. Another potential source of uncertainty when comparing past and current distribution of species is related to changes in available information. The increase in sampling effort and the use of more advanced techniques in the last few decades has helped to improve the knowledge of species distributions; consequently, some changes in the maps might be due to an over- or underestimation of past ranges. Taxonomic changes are another issue when attempting to compare past and present distributions of species, and have been a limitation to our sample size. From 1993 to 2008, almost 1000 new species have been described^48^, and since the Red List assessment in 2008, 561 species experienced further taxonomic changes^6^. This implies that, for many of the currently recognised species, there have been major challenges in delineating past distributions. However, our sample includes both slow and fast life histories (Supplementary Fig. 9) to ensure the relevance of our results across terrestrial non-volant mammals. Finally, we could not include hunting as a variable in our models, despite it representing a major threat for several large mammals; the variance that remained unexplained in our models is probably related to this missing variable.

By identifying the characteristics that make species more or less resilient to human pressure, it is possible to anticipate future range loss and prevent further species’ declines. Species with low reproductive rates require particular conservation attention in this context, as they are more likely to suffer from range contractions and less likely to colonise new areas, compared with other species. Although they may be potentially capable, these species are unlikely to succeed in colonising, or recolonising, new areas without conservation intervention. Among these species, those with small ranges require careful management of the areas surrounding their remaining distributions, the creation of corridors and/or assisted colonisation. This is especially important in light of the current and future effects of climate change^49^. As threats associated with climate change increase in severity, many mammal species may be less able to move towards unoccupied suitable areas. Our results also highlight the interactions between life history and anthropogenic pressures to determine range changes, thus suggesting that management solutions should take into account both threats and intrinsic characteristics of species.

## Methods

### Current and past distribution ranges

We used species range maps from the 1970s^24^ and the present day, following IUCN Red List taxonomy, to measure the past change in species’ distributions. We represented the current distribution of terrestrial mammal species using the most recent maps of geographical ranges from the IUCN Red List of Threatened Species^6^, excluding all human-mediated introductions, and the reintroductions that occurred after 1970. Similar to current IUCN distribution ranges, all the maps we collected for the 1970s are expert-based. We included in the analysis only those species’ maps produced following mapping protocols reflecting current IUCN Red List standards^6^, and for which the 1970s distribution was compatible with textual descriptions of the past range in the recent literature^24^. Distribution ranges were available from the literature as polygons, with the exception of Australian maps, for which we extrapolated occurrence records from range maps in the last action plan of Australian mammals^9^. This action plan contains distributional data that the experts collated from individual datasets maintained by museums and government conservation agencies of all Australian states and territories. For each of the 204 mammal species, a polygonal map of the range was created using all distributional information available, along with knowledge of habitat, elevation limits and other expert knowledge of the taxon, following IUCN^6^. These polygons display the limits of each species’ distribution. For non-Australian species, maps have been revised by a certified IUCN Red List Assessor, M.P., and the Coordinator of the Global Mammal Assessment for IUCN, C.R. Australian mammals have been revised by A.A.B. and J.C.Z.W., members of the IUCN Species Survival Commission (SSC) Australian Marsupials and Monotremes Specialist Group, responsible for the IUCN Red List assessments of Australian mammals, and lead authors of the Australian mammals action plan. We looked at literature evidence to support changes observed between past and present ranges, to make sure that any difference in distribution derived from a real change, and was not an artefact of improved knowledge. In order to avoid uncertainty related to improved information on species’ distribution, we disregarded non-genuine changes in Red List status associated with changes in species’ ranges. For example, the 1970s maps of *Herpestes sanguineus* we found in the literature included most of Sub-Saharan Africa, with the exception of southern Africa and Western Namibia. However, it seems that the species today is only patchy distributed in Gabon, marginally in Congo, South Africa and Equatorial Guinea, and lost vast portions of its range in Cameroon, Democratic Republic of Congo, Nigeria and Central African Republic^50^, but we could not find enough evidence in the literature to support these reductions. Hence, we decided to exclude this species and many others with similar uncertainties from the database. Another example of species excluded from our data is *Sus cebifrons*. This species is endemic to the West Visayan Islands in the Philippines. The species is currently classified as Critically Endangered and declining^51^, but its range in Panay, the island with the largest population, is bigger than the one we found for the 1970s. Since it is not realistic, we decided to exclude the species from the analysis. We georeferenced and digitised all the maps with QGIS v3.2 to create vector data.

We obtained past distribution ranges for a total of 204 species. Although this sample represents only about 5% of all terrestrial non-volant mammals, it includes representatives of all major taxonomic orders and all terrestrial realms (Supplementary Table 1). In addition, we checked for inclusion of extreme values in the life-history predictors by comparing the probability-density functions of body mass and weaning age (the traits with the highest predictive importance in our models) in our sample and in all terrestrial non-volant mammals (Supplementary Fig. 9).

### Distribution range comparison

In order to measure the maximum possible range expansion of the species in the study period, we created a buffer around the past range of each species, depicting the area that the species could theoretically colonise through dispersal during the study period (Supplementary Fig. 1)

1 $${\mathrm{BR}} = D * \left( {{\it{\Delta}} t/{\mathrm{AFR}}} \right),$$

where BR is the buffer radius (in km), *D* is the species dispersal capacity (in km), *Δt* is the time interval of the study period (in years) and AFR is the species age at first reproduction (in years). Dispersal values were calculated with the formula provided by Santini et al.^30^, and age at first reproduction estimates was mainly obtained from PanTHERIA^52^ and AnAge^53^ database, following the methods used in Pacifici et al.^54^ This allowed us to quantify how much of the potential range expansion—within the buffer around past distribution range—was realised by each species. By using age at first reproduction, there was no species whose current range went beyond the potential colonisation buffer around the past range. To be more conservative in terms of species’ potential colonisation, we also performed a sensitivity analysis by using generation-length estimates (from Pacifici et al.^54^) instead of age at first reproduction. This approach represents a more conservative colonisation scenario where members of one of the litters produced by an individual during its reproductive lifespan will disperse in a given direction, and members of one of their litters will do the same. In repeating our analyses under both scenarios, we showed that our results are robust to uncertainty associated with estimates of species’ colonisation potential. We measured range expansion as the percentage of area gained within the buffer area (Supplementary Data 1). This allowed to measure, for each species, the condition of habitats in areas that could be potentially reached by the species.

Range contraction was computed by calculating the percentage of area lost with respect to the past range (Supplementary Data 1). In order to identify proportional change in range size, we also computed the percentage of area gained with respect to the past range (Supplementary Data 1). Our approach allowed us to include the same set of species, both in the range contraction and in the range expansion model, quantifying extrinsic variables within the past range and the potential expansion buffer for all species; this made the two models fully comparable with each other.

### Human drivers of range change and other extrinsic variables

To account for land-use changes in the study period, we used a set of past data based on HYDE from the Land-Use Harmonisation phase 2 project (http://luh.umd.edu/). The land-use harmonisation past dataset covers the period 1850–2015, and includes annual proportions of 12 land-cover types calculated within 0.25° × 0.25° cells. We extracted mean values of these proportions for urban lands and natural areas for the time period 1960–1980 to cover the time interval considered for the past ranges. For the present, we used mean values for the period 2008–2015 to cover the time interval of current ranges (i.e. post 2008 IUCN Red List global mammal assessment). The latter was obtained by summing the proportion of natural vegetation variables available in the dataset, i.e. primarily forested and non-forested lands. We used these data to calculate the difference in the proportion of both natural and urban land between the 1970s and the present. Because for many species one of the main issues that prevented expansion was the presence of heavily modified and urbanised environments surrounding the distribution range, we decided to also consider the proportion of urban land in the past as a stand-alone variable.

Data related to temperature and precipitation variation were taken from the Climatic Research Unit database version 4.01. This database covers the period 1901–2016 with monthly values for mean temperature and precipitation. For the past, we obtained mean values of these variables for the time interval 1960–1980 in order to express, with an acceptable approximation, the average climatic values for the study period. Current climate was derived from the time interval 2012–2016, in order to reflect the years of the ongoing global mammal IUCN Red List reassessment. Then, we computed the difference between the current and past climate in order to identify the areas where these changes have been more severe, and their directions (increase or decrease). We selected these climatic variables in order to test two different hypotheses: (1) stable or similar climate favours range expansion and (2) dissimilar climate is related to range contraction^11,55,56^.

Other proxies of human pressure include the presence of buildings and human population density. These factors can be considered not only as direct stressors for species, but also as indirect indicators of changes in the landscape that we could not include as variables in our models due to a lack of data (e.g. resource extraction). The Global Human Settlement (GHS) is a multi-temporal information layer on built-up presence, as derived from Landsat image collections (GHS1975 for the past and ad hoc Landsat 8 collection 2013/2014 for the present^57^) at a resolution of 1 km. Residential population estimates, expressed as the number of people per cell, for target years 1975 and 2015, were developed by the European Commission Joint Research Centre. As for land cover and climatic variables, we computed the difference between the proportion of buildings and the human population density per cell in the present and in the 1970s, but used the human population density in 1975 also as a stand-alone variable.

Invasive alien species are the second most common threat associated with species’ extinction since AD 1500, and they have been the major driver of population decline and range collapse for Australian mammals^9^. This might lead to inherent differences in the correlates of range contraction and expansion between Australian mammals and mammals from other continents. However, free access to information on the current distribution of introduced species remains challenging^58^, and it was not possible to find reliable fine-resolution data for these species in the past. In order to identify whether species in different geographic contexts had different responses to human pressure, we used the biogeographic realm as a predictor in our models. Finally, to control for the fact that catastrophic events are less likely to impact large populations than smaller ones, we included the size of the 1970s range as a predictor in our models.

### Life-history traits

For our analysis, we used a database of mammal life-history traits, which includes data from different sources^52,53,59^. These are (1) body mass (grams), which refers to the mean mass of adult individuals; (2) diet breadth, the number of categories of food items eaten by a species; (3) dispersal distance (km), the maximum distance covered by young individuals when they move from their birth to the breeding site; (4) generation length (days), the average age of parents of the current cohort; (5) litter size, the mean number of offspring born per litter; (6) litters per year, the mean number of litters per female per year; (7) habitat specificity, which refers to the number of habitats in which a species has been recorded (raw data downloaded from www.iucn.org); (8) weaning age (days), considered as the age at which the offspring no longer receives milk from the mother and independent foraging begins to make a major contribution to its energy requirements. To consider additional aspects not included in the life-history traits considered, but characterising different taxonomic groups, we included taxonomic order as a categorical variable in our models. We had six taxonomic orders, including just one or two species each, (Supplementary Table 1) and they were all small mammals; therefore, we decided to group them together in the Other group, with a total of 11 final levels for the Order variable. We did a test by including phylogenetic eigenvectors as variables in our random forest models to see if there was a phylogenetic signal in the responses. We represented species’ phylogeny by extracting phylogenetic eigenvectors^60^ from the PHYLACINE dataset^61^. We tested the use of an incremental number of eigenvectors (5, 10 and 20) as model predictors, following ref. ^62^. In all cases, we always found a decrease in the percentage variance explained by the Random Forest models that included the eigenvectors, compared with the models without phylogeny. This was verified both for the range contraction and range expansion models. Therefore, we did not consider the phylogenetic signal in our final models.

### Statistical analyses

We used Random Forest regression models^27^ implemented in the R package randomForest to assess the role of life-history traits and extrinsic factors in predicting range changes for terrestrial non-volant mammals. Random Forest is a machine-learning technique often used in ecological analyses^16,63^ that combines hundreds of regression trees (1000 trees in our analyses); these trees carry out a recursive binary partitioning of the response variable to create groups that are increasingly homogeneous^64^. In fitting a regression tree, a random forest model carries out an optimisation to select a node, together with a predictor variable and a predictor cut-off, in order to obtain the best split of the response variable^27^. One of the outputs of random forest models is the estimate of the relative importance of each independent variable in predicting the response variable, which is obtained by measuring the relative increase in the model mean square error when the variable values are randomly permuted. Random Forest is a well-established analytical technique for analysing mammal species’ response to a mix of variables^65,66^.

Instead of doing a single range-change model, which would have prevented us from assessing the different role of predictors in areas gained and lost, we built two separate models. In the first model (hereafter the range contraction model), the response variable was the percentage of past distribution range lost by each species. The values of spatial predictor variables for the range contraction model, resampled at 10- and 100-km resolution, were computed within the 1970s range of the species (e.g. human population density within the species’ past range). In the second model (hereafter the range expansion model), the response variable was the percentage of area gained within the buffer. In this case, the values of spatial predictor variables were extracted from within the buffer around the past range (e.g. human population density in areas surrounding the species’ past range).

We measured the percentile distribution of human-pressure variables within species’ past ranges (and their buffers), to represent the extent of high-pressure levels^67^. In order to select the most explanatory percentiles, we ran different restricted Random Forest models where the only predictors were the percentiles of each external driver, and checked the relative variable importance for both the contraction and expansion models. For each cell, we extracted the value of the anthropogenic drivers, and computed the 5th, 10th, 50th, 90th and 95th percentile of the distribution of each predictor, in both the lost portions of the past range and the gained areas within the buffer. These percentiles have been tested in order to find the one with the highest correlation with the response variables. We then used the best percentiles to run two full Random Forest models (for range contraction and expansion) that included both extrinsic and life-history variables. We considered change as a loss or gain >5% in the distribution range.

We used the package plotmo in R^68^ to create partial dependence plots to show the effects of pairs of selected predictors on the response variable.

### Model selection and validation

We followed the methodology developed by Murphy et al.^69^ for metric selection based on optimal model fit with the lower number of metrics. Although Random Forest models can operate with large numbers of variables, this can lead to an increase in the correlation of trees, reducing the overall performance of the model. One of the outputs of a Random Forest is the order of importance of each variable (I) based on the number of times a given variable decreased mean-squared error (MSE). For both the contraction and expansion models, we ran an initial model with all variables, and then calculated a model improvement ratio (MIR) for each variable. The MIR metric, unlike the raw I score, is not influenced by the total number of variables in the model, and is comparable among models. MIR is calculated as [*I*_*n*_/*I*_max_], where *I*_*n*_ is the importance of a given variable, and *I*_max_ is the maximum model improvement score. By using the R package rfUtilities^70^, we iterated through MIR thresholds (0–1, in 0.1 increments), retaining all variables above the given threshold. The algorithm selects the threshold that minimised retained variables, model MSE and maximised the percentage of variation explained. We also obtained a series of validation statistics: MSE, root MSE and mean absolute error (Supplementary Figs. 7 and 8).

### Reporting summary

Further information on experimental design is available in the Nature Research Reporting Summary linked to this paper.

## Supplementary information

Supplementary Information

Supplementary Data 1

Reporting Summary

## Data Availability

Data sources for past ranges are available at https://onlinelibrary.wiley.com/doi/10.1002/ecy.2747/suppinfo. IUCN current ranges are available upon request at https://www.iucnredlist.org/. Population density and the proportion of buildings’ data are available at https://ghsl.jrc.ec.europa.eu/datasets.php. Land-use data are available at https://luh.umd.edu/data.shtml. Climatic data are available at http://www.cru.uea.ac.uk/data. PanTHERIA database is available at 10.1890/08-1494.1. AnAge database is available at https://genomics.senescence.info/species/. Current ranges not taken from the IUCN Red List for sensitive species are available from the corresponding author upon reasonable request.
