# Supplementary material for: Global correlates of range contractions and expansions in terrestrial mammals
Source: Nat Commun. 2020 Jun 5;11:2840. doi: 10.1038/s41467-020-16684-w (PMC7275054; doi:10.1038/s41467-020-16684-w)
Supplement: Supplementary file 1 — Supplementary Information [file 41467_2020_16684_MOESM1_ESM.pdf]

TITLE: Global correlates of range contractions and expansions in terrestrial mammals

Pacifici et al.

Supplementary Table 1 Number of species in the analyses by taxonomic order

Supplementary Table 2 Acronyms used for the variables in loss model.

Supplementary Table 3 Acronyms used for the variables in the expansion model.

Supplementary Figure 1 Creation of the buffer around the past range.

Supplementary Figure 2 Plot showing the relationship between the percentage of area gained (y axis) and the percentage of area lost (x axis) by each species.

Supplementary Figure 3 Partial dependence plots for range contraction (10 km resolution).

Supplementary Figure 4 Sensitivity analysis showing random forest variable importance plot for range contraction.

Supplementary Figure 5 Sensitivity analysis showing random forest variable importance plot for range expansion with buffer calculated with generation length.

Supplementary Figure 6 Partial dependence plots for range expansion.

Supplementary Figure 7 Validation statistics for the best model for range contraction.

Supplementary Figure 8 Validation statistics for the best model for range expansion.

Supplementary Figure 9 Frequency distribution of Weaning Age (WA) and log Body Mass (BM).

SUPPLEMENTARY TABLE 1 Number of species in the analyses by taxonomic order

**Taxonomic Order**	***n* species**
Afrosoricida	1
Carnivora	52
Cetartiodactyla	23
Cingulata	2
Dasyuromorphia	6
Didelphimorphia	2
Diprotodontia	7
Eulipotyphla	6
Lagomorpha	8
Macroscelidea	1
Microbiotheria	1
Peramelemorphia	2
Perissodactyla	6
Primates	19
Proboscidea	2
Rodentia	66

SUPPLEMENTARY TABLE 2 Acronyms used for the variables in the contraction model.

**Variable acronym**	**Description**
D_urb_p_50	Difference in the proportion of anthropic classes of land use (50 percentile) between the present and the past, positive values indicate an increase in the proportion of anthropic land use
D_nat_p_90	Difference in the proportion of natural classes of land use (90 percentile) between the present and the past, positive values indicate an increase in the proportion of natural land use
D_build_p_95	Difference in the proportion of buildings (95 percentile) between the present and the past, positive values indicate an increase in the proportion of buildings
urb_1970_p_95	Proportion of anthropic classes of land use within a cell (95 percentile) in the past
D_pop_p_95	Difference in the density of human population (95 percentile) between the present and the past, positive values indicate an increase in density
D_pre_p_95	Difference in mean annual precipitation (95 percentile) between the present and the past, positive values indicate an increase in precipitation
D_tmp_p_50	Difference in mean annual temperature (50 percentile) between the present and the past, positive values indicate an increase in temperature
pop_1975_p_95	Human population density (95 percentile) in the past
BM	Body mass
Diet	Dietary breadth
Order	Taxonomic order
Gen_length	Generation length
Habitat_cat	Habitat breadth
LS	Litter size
LY	Litters per year
WA	Weaning age
Realm	Biogeographic realm
tot_past	Historic range size in km^2

SUPPLEMENTARY TABLE 3 Acronyms used for the variables in the expansion model. Intrinsic traits acronyms same as in the contraction model (Supplementary Table 2).

**Variable acronym**	**Description**
D_nat_b_50	Difference in the proportion of natural classes of land use (50th percentile) between the present and the past computed within the buffer, positive values indicate an increase in the proportion of natural land use
D_urb_b_50	Difference in the proportion of anthropic classes of land use (50th percentile) between the present and the past computed within the buffer, positive values indicate an increase in the proportion of anthropic land use
urb_1970_b_90	Proportion of anthropic classes of land use within a cell (90th percentile) in the past computed within the buffer
D_pop_b_90	Difference in the density of human population (90th percentile) between the present and the past computed within the buffer, positive values indicate an increase in density
D_pre_b_50	Difference in mean annual precipitation (50th percentile) between the present and the past computed within the buffer, positive values indicate an increase in precipitation
D_tmp_b_10	Difference in mean annual temperature (10th percentile) between the present and the past computed within the buffer, positive values indicate an increase in temperature
pop_1975_b_95	Human population density (95th percentile) in the past computed within the buffer
D_build_b_90	Difference in the proportion of buildings (90th percentile) between the present and the past computed within the buffer, positive values indicate an increase in the proportion of buildings

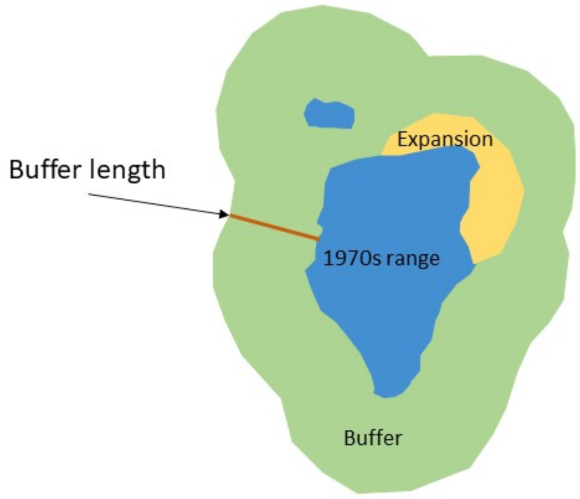

SUPPLEMENTARY FIGURE 1 Creation of the buffer around the past range. The blue polygon represents the past range of the species, the green area represents the area that the species could potentially colonise during the study period, and the yellow polygon is the area actually colonised. See also equation (1) in the main text.

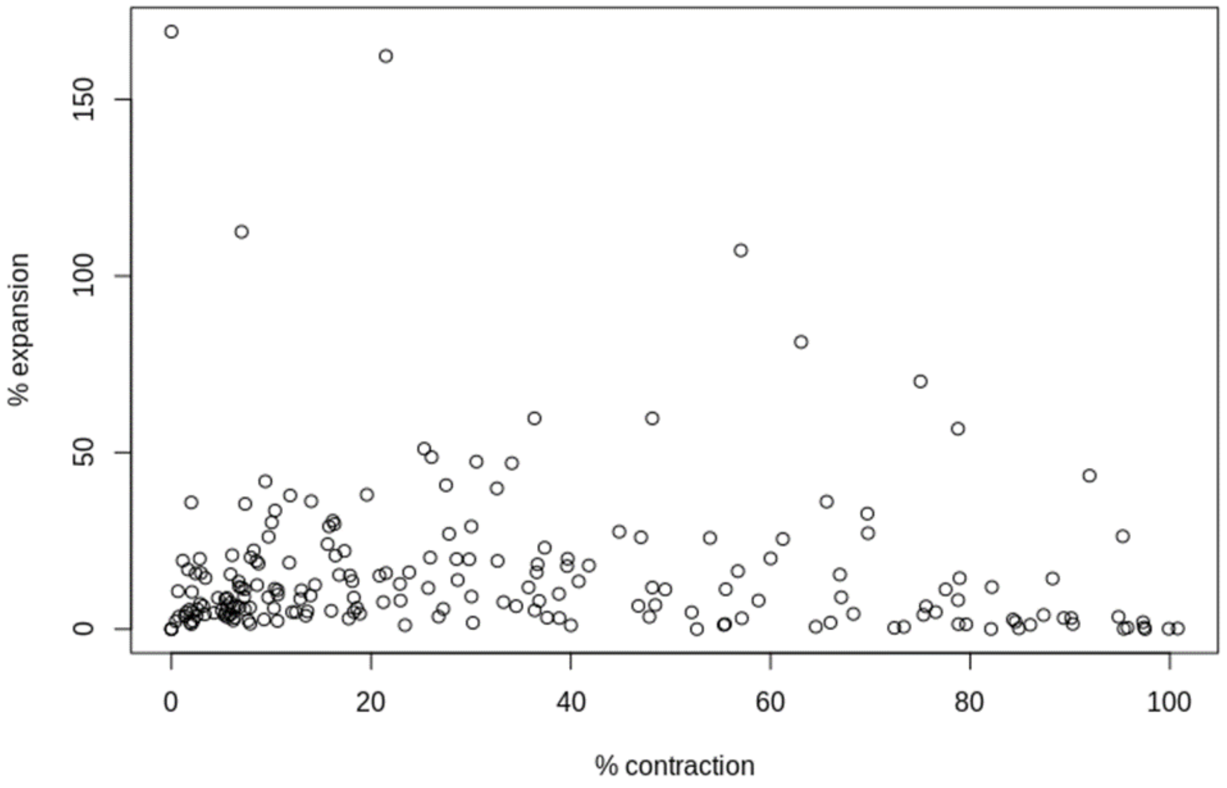

SUPPLEMENTARY FIGURE 2 Plot showing the relationship between the percentage of area gained (y axis) and the percentage of area lost (x axis) by each species. n=204 species have been analysed.

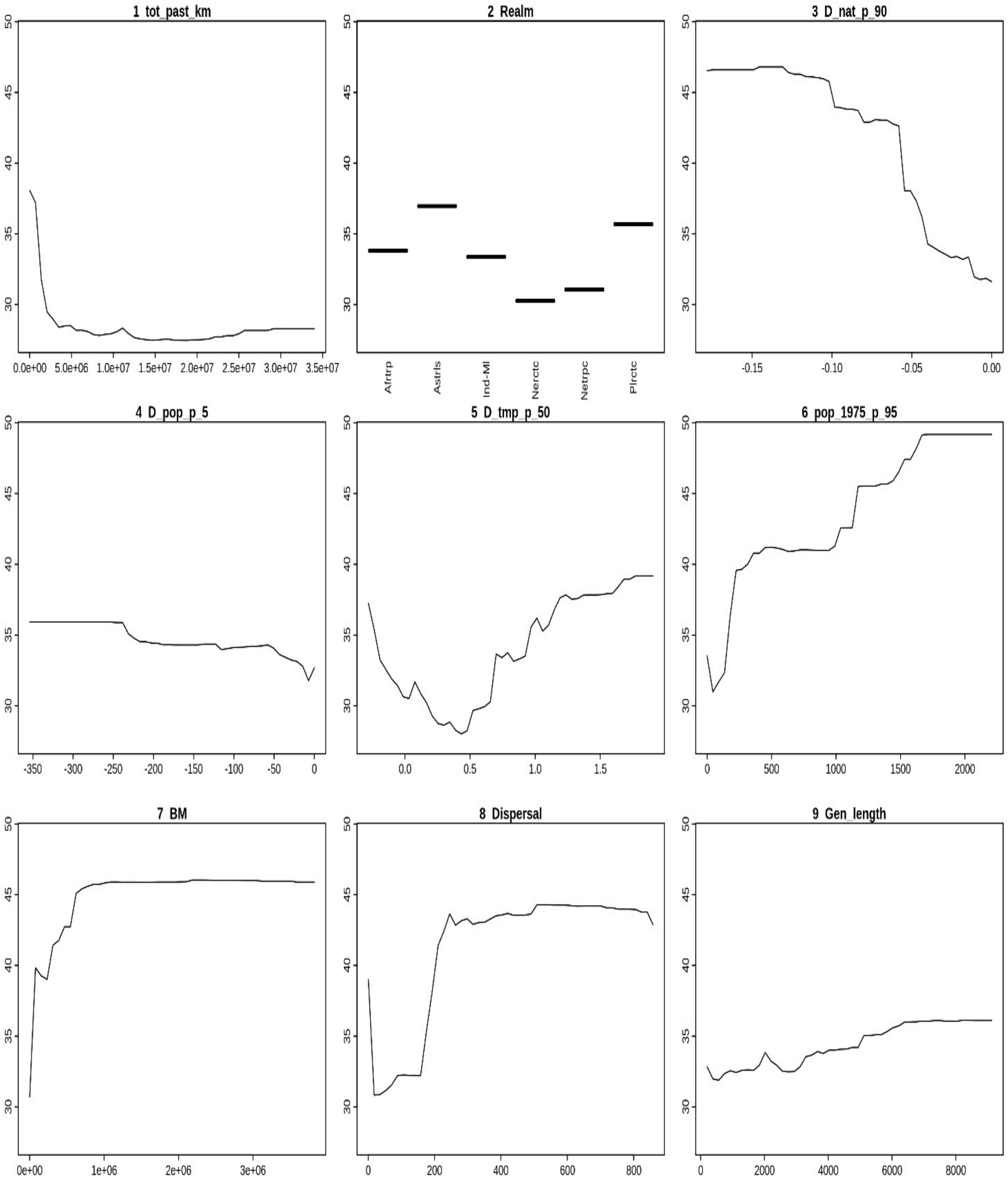

SUPPLEMENTARY FIGURE 3 Partial dependence plots for range contraction (10 km resolution). The plots represent the relationship between each predictor (x-axis) and the response variable (% range loss, y-axis). See Supplementary Table 2 for acronyms.

Bars in the Realm graph represent Afrotropic, Australasia, Indo-Malay, Nearctic, Neotropic and Palearctic realms, respectively.

Y axis has been standardised in all graphs to reflect the relative influence of each predictor on the dependent variable.

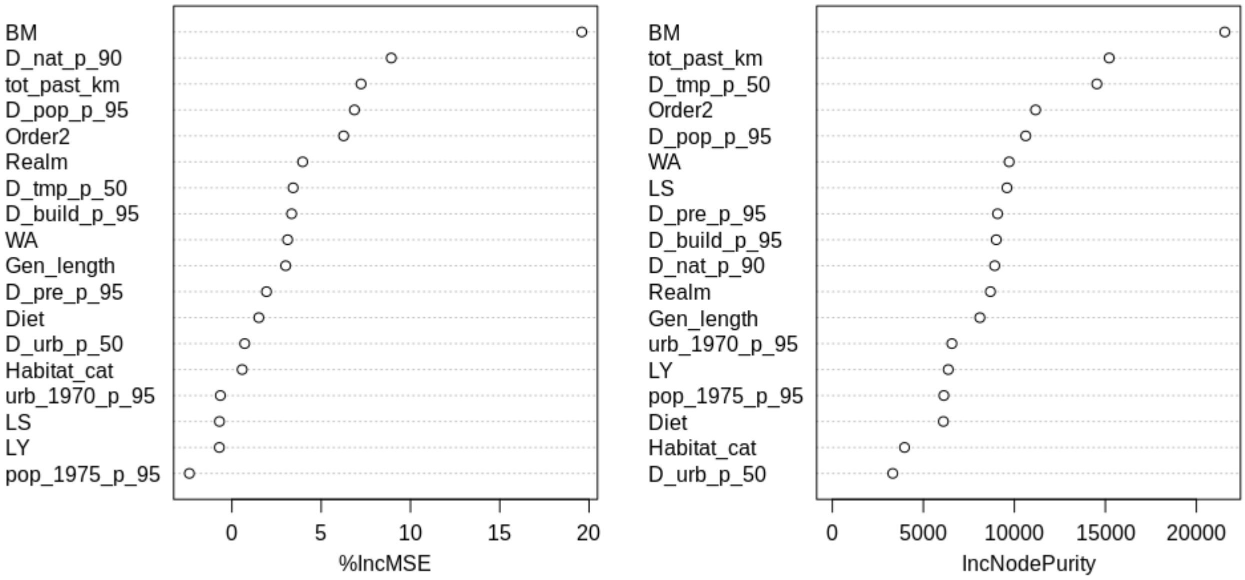

SUPPLEMENTARY FIGURE 4 Sensitivity analysis showing random forest variable importance plot for range contraction. The plot shows the decreasing importance of intrinsic and extrinsic variables at 100 km resolution in predicting proportional loss in species range. Plots how much MSE (%IncMSE) or Impurity (IncNodePurity) increase when a variable is randomly permuted. Big changes in these measures indicate important variables. n=204 species have been analysed. See Supplementary Table 2 for acronyms.

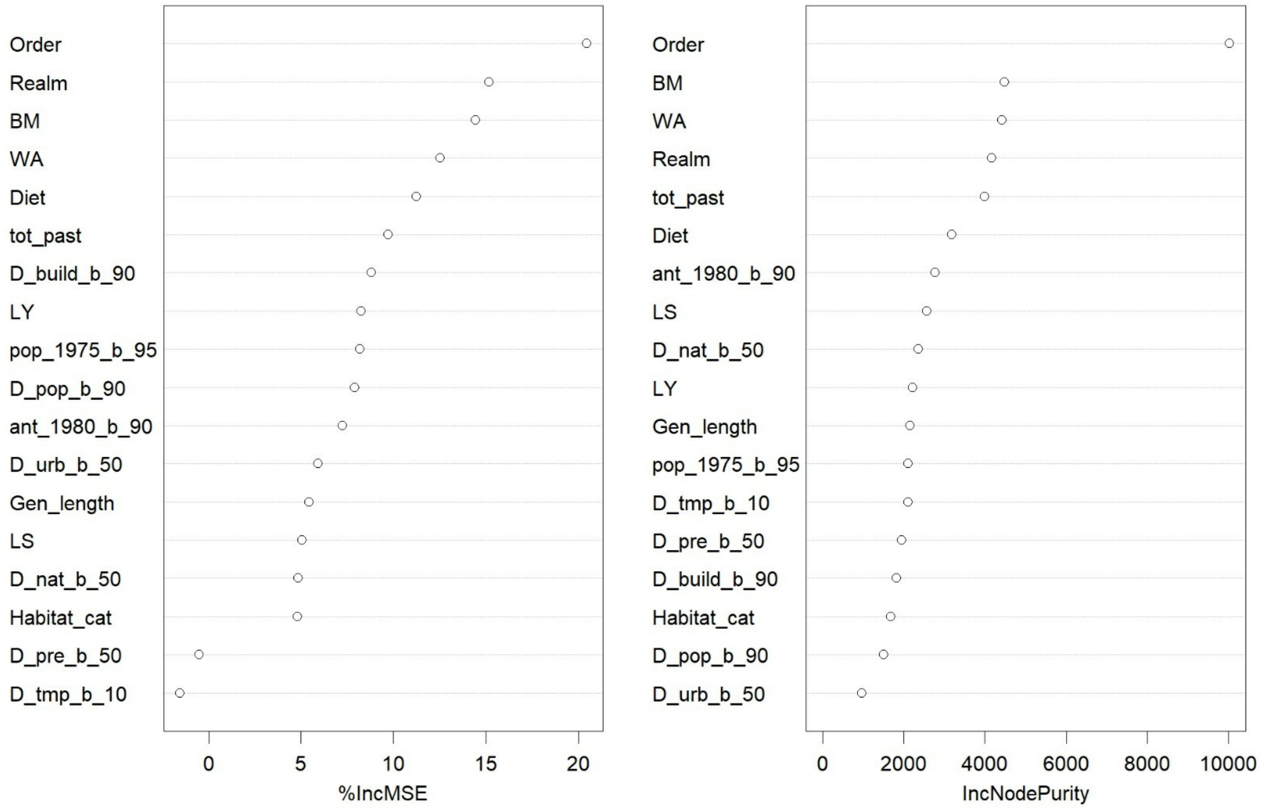

SUPPLEMENTARY FIGURE 5 Sensitivity analysis showing random forest variable importance plot for range expansion with buffer calculated with generation length**.** Plots how much Mean Square Error (%IncMSE) or Impurity (IncNodePurity) increase when a variable is randomly permuted. Big changes in these measures indicate important variables. n=204 species have been analysed. See Supplementary Tables 2, 3 for acronyms.

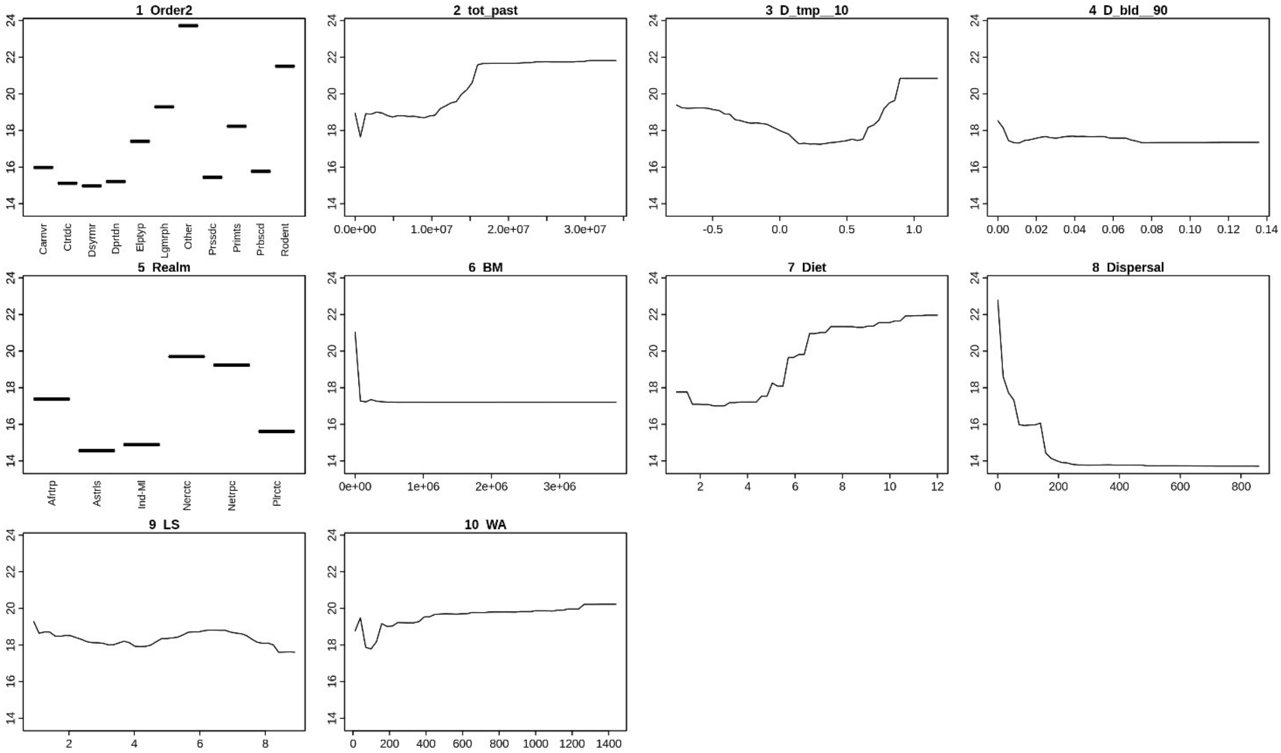

SUPPLEMENTARY FIGURE 6 Partial dependence plots for range expansion. The plots represent the relationship between each predictor (x-axis) and the response variable (partial dependence on % range expansion within the buffer, y-axis). n=204 species have been analysed. See Supplementary Tables 2, 3 for acronyms.

Bars in the Order graph represent Carnivora, Cetartiodactyla, Dasyuromorphia, Diprotodontia, Eulipotyphla, Lagomorpha, Other (including Afrosoricida, Cingulata, Didelphimorphia, Macroscelidea, Microbiotheria, Peramelemorphia), Perissodactyla, Primates, Proboscidea, Rodentia, respectively.

Bars in the Realm graph represent Afrotropic, Australasia, Indo-Malay, Nearctic, Neotropic and Palearctic realms, respectively.

Y axis has been standardised in all graphs to reflect the relative influence of each predictor on the dependent variable.

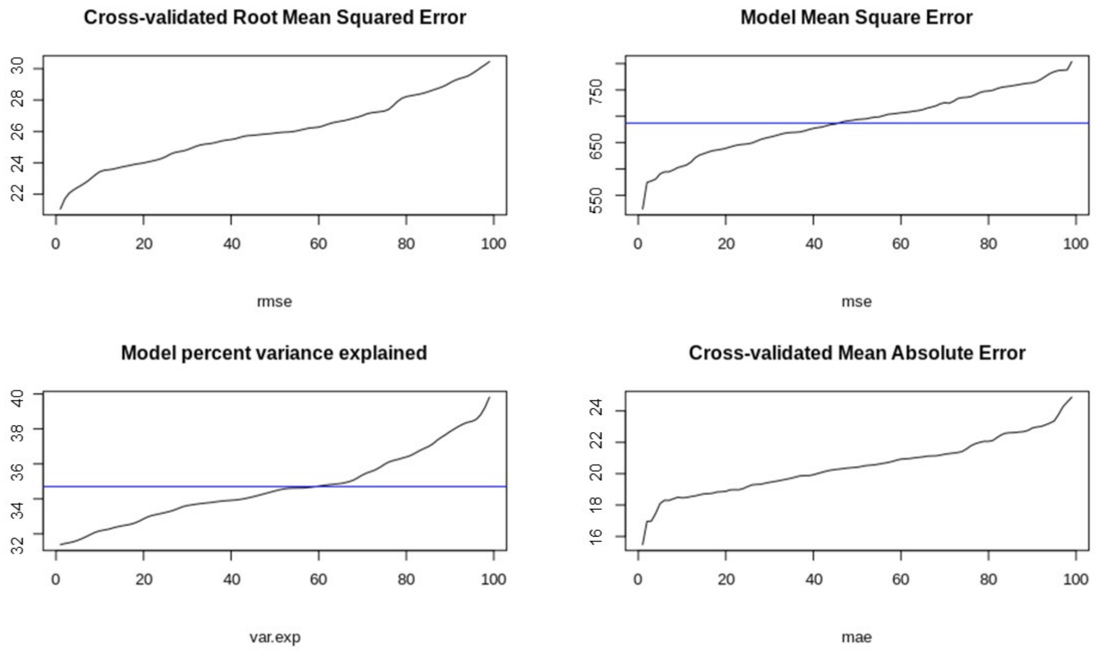

SUPPLEMENTARY FIGURE 7 Validation statistics for the best model for range contraction.

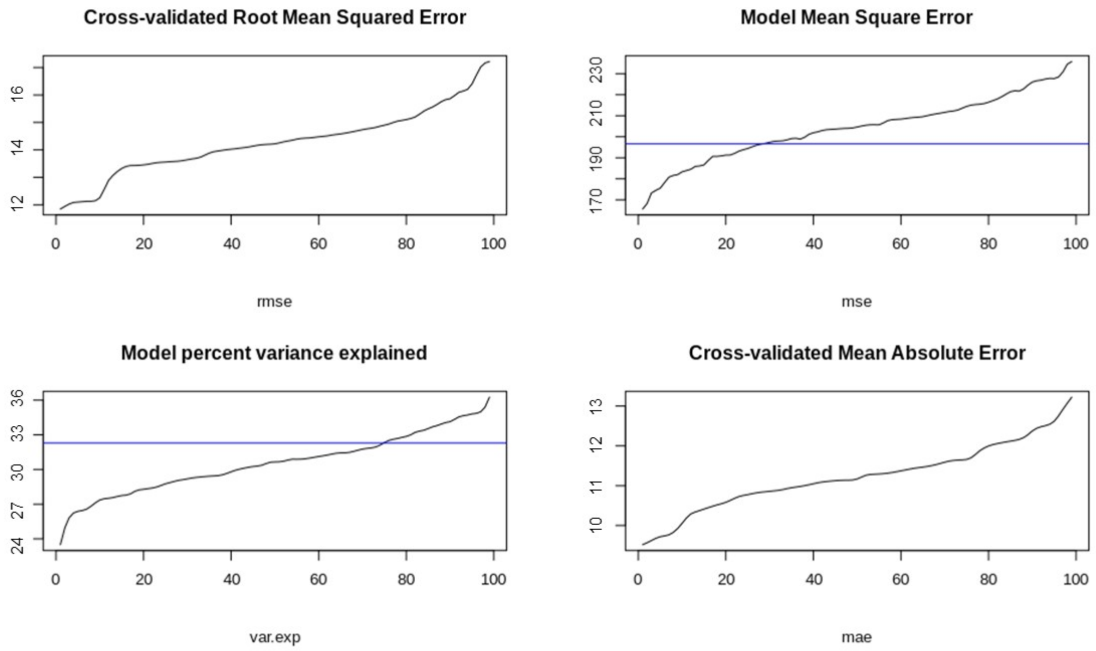

SUPPLEMENTARY FIGURE 8 Validation statistics for the best model for range expansion.

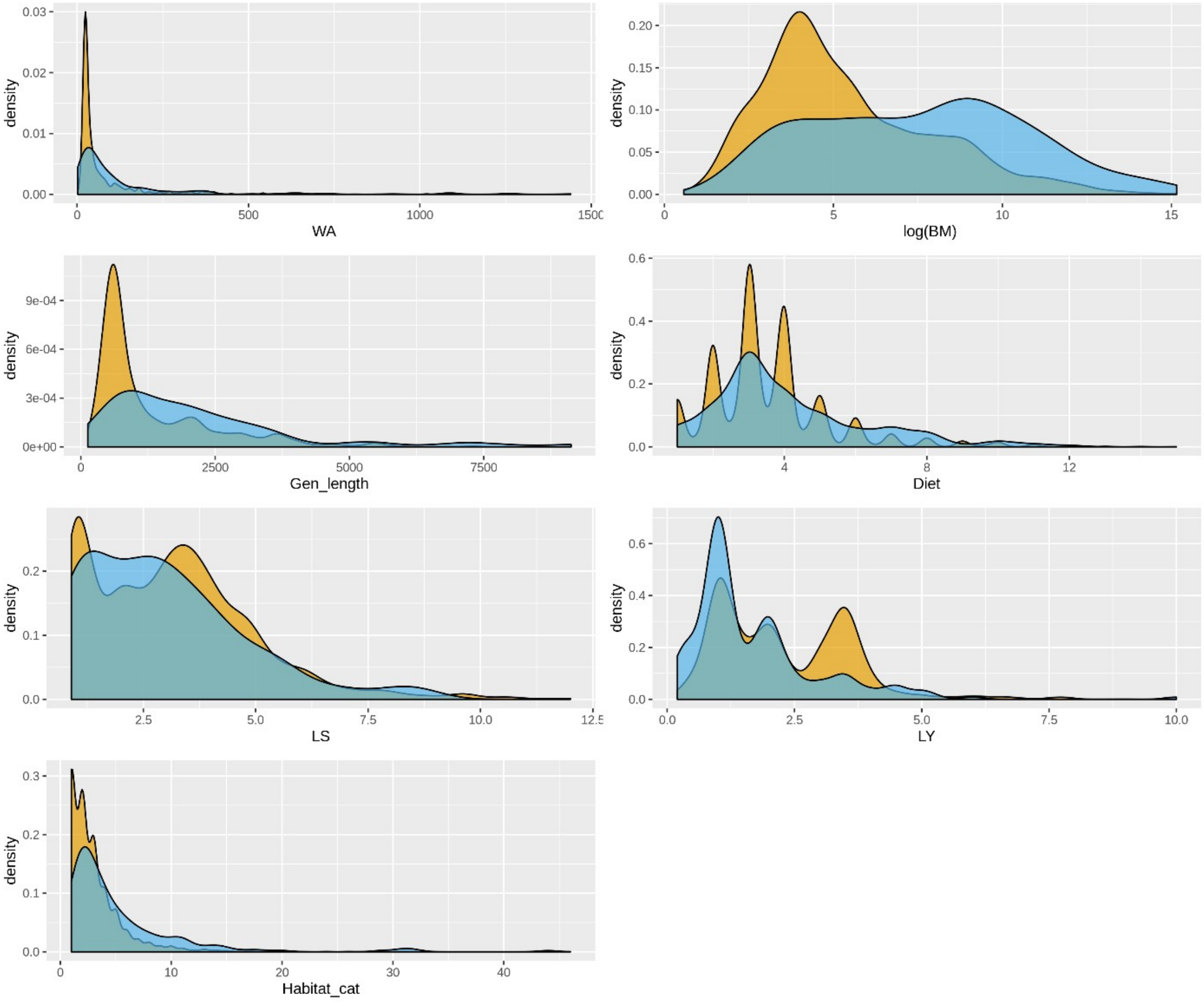

SUPPLEMENTARY FIGURE 9 Frequency distribution of life-history traits. Yellow areas under the curve represent the distribution of values for the two variables in all terrestrial non-volant mammals, while blue areas show the distribution in our sample. See Supplementary Tables 2, 3 for acronyms.
