# Supplementary material for: Global correlates of range contractions and expansions in terrestrial mammals
Source: Nat Commun. 2020 Jun 5;11:2840. doi: 10.1038/s41467-020-16684-w (PMC7275054; doi:10.1038/s41467-020-16684-w)
Supplement: Supplementary file 3 — Reporting Summary [file 41467_2020_16684_MOESM3_ESM.pdf]

nature research

Corresponding author(s): Michela Pacifici

Last updated by author(s): May 5, 2020

Reporting Summary

Nature Research wishes to improve the reproducibility of the work that we publish. This form provides structure for consistency and transparency in reporting. For further information on Nature Research policies, see Authors & Referees and the Editorial Policy Checklist .

Statistics

For all statistical analyses, confirm that the following items are present in the figure legend, table legend, main text, or Methods section.

n/a	Confirmed	
☐	☒	The exact sample size (*n*) for each experimental group/condition, given as a discrete number and unit of measurement
☒	☐	A statement on whether measurements were taken from distinct samples or whether the same sample was measured repeatedly
☐	☒	The statistical test(s) used AND whether they are one- or two-sided<br>*Only common tests should be described solely by name; describe more complex techniques in the Methods section.*
☐	☒	A description of all covariates tested
☐	☒	A description of any assumptions or corrections, such as tests of normality and adjustment for multiple comparisons
☒	☐	A full description of the statistical parameters including central tendency (e.g. means) or other basic estimates (e.g. regression coefficient) AND variation (e.g. standard deviation) or associated estimates of uncertainty (e.g. confidence intervals)
☒	☐	For null hypothesis testing, the test statistic (e.g. *F*, *t*, *r*) with confidence intervals, effect sizes, degrees of freedom and *P* value noted<br>*Give P values as exact values whenever suitable.*
☒	☐	For Bayesian analysis, information on the choice of priors and Markov chain Monte Carlo settings
☒	☐	For hierarchical and complex designs, identification of the appropriate level for tests and full reporting of outcomes
☒	☐	Estimates of effect sizes (e.g. Cohen's *d*, Pearson's *r*), indicating how they were calculated

Our web collection on statistics for biologists contains articles on many of the points above.

Software and code

Policy information about availability of computer code

Data collection	No software was used for data collection
Data analysis	R software version 3.6.3 for statistical analysis, QGIS version 3.4 and GRASS GIS7.8.0 for GIS analysis (all software are open source)

For manuscripts utilizing custom algorithms or software that are central to the research but not yet described in published literature, software must be made available to editors/reviewers. We strongly encourage code deposition in a community repository (e.g. GitHub). See the Nature Research guidelines for submitting code & software for further information.

Data

Policy information about availability of data

All manuscripts must include a data availability statement. This statement should provide the following information, where applicable:

- Accession codes, unique identifiers, or web links for publicly available datasets
- A list of figures that have associated raw data
- A description of any restrictions on data availability

Data sources for past ranges are available at https://onlinelibrary.wiley.com/doi/10.1002/ecy.2747/suppinfo. IUCN current ranges are available upon request at https://www.iucnredlist.org/. Population density and proportion of buildings data are available at https://ghsl.jrc.ec.europa.eu/datasets.php. Land use data are available at https://luh.umd.edu/data.shtml. Climatic data are available at http://www.cru.uea.ac.uk/data. PanTHERIA database is available at https://doi.org/10.1890/08-1494.1. AnAge database is available at https://genomics.senescence.info/species/. Current ranges not taken from the IUCN Red List for sensitive species are available from the corresponding author upon reasonable request.

Field-specific reporting

Please select the one below that is the best fit for your research. If you are not sure, read the appropriate sections before making your selection.

☐ Life sciences ☐ Behavioural & social sciences ☒ Ecological, evolutionary & environmental sciences

For a reference copy of the document with all sections, see nature.com/documents/nr-reporting-summary-flat.pdf

Ecological, evolutionary & environmental sciences study design

All studies must disclose on these points even when the disclosure is negative.

Study description	Analysis of intrinsic and extrinsic factors that influenced changes in mammals distribution in the past 50 years
Research sample	A set of 204 terrestrial non-volant mammals, including all major taxonomic orders, for which 1970s distributions were present in the Pacifici et al. 2019 paper in Ecology (https://doi.org/10.1002/ecy.2747)
Sampling strategy	Species were selected for the analysis based on availability of past distributions in the Pacifici et al. 2019 paper in Ecology (https://doi.org/10.1002/ecy.2747). All species in the above-mentioned dataset have been included in the analysis
Data collection	Michela Pacifici and Andrea Cristiano used data on historic ranges on mammals present in the Pacifici et al. 2019 paper in Ecology (https://doi.org/10.1002/ecy.2747). For present ranges, IUCN Red List range maps have been collected and downloaded from the IUCN Red List website (www.iucnredlist.org)
Timing and spatial scale	The time period considered for the analysis is 1970s-2017. We used this timeframe because human activities have steeply intensified in these decades. We stopped at 2017 because it is the last date of availability for the anthropogenic variables used. The study includes distribution ranges of the species analysed at a global scale at 10 km resolution
Data exclusions	We included all maps present in the Pacifici et al. 2019 paper (https://doi.org/10.1002/ecy.2747). In this paper, the authors excluded those maps for the past that did not follow IUCN mapping standards or that were considered unreliable because the changes in distribution were not supported by scientific literature.
Reproducibility	All the variables used are freely available, both the maps and intrinsic and extrinsic predictos
Randomization	We used a Random Forest model, which builds multiple decision trees and combines the results to get a more accurate prediction.
Blinding	Blinding was not relevant since we collected data from the literature

Did the study involve field work? ☐ Yes ☒ No

Reporting for specific materials, systems and methods

We require information from authors about some types of materials, experimental systems and methods used in many studies. Here, indicate whether each material, system or method listed is relevant to your study. If you are not sure if a list item applies to your research, read the appropriate section before selecting a response.

Materials & experimental systems

n/a	Involved in the study
☒	☐ Antibodies
☒	☐ Eukaryotic cell lines
☒	☐ Palaeontology
☒	☐ Animals and other organisms
☒	☐ Human research participants
☒	☐ Clinical data

Methods

n/a	Involved in the study
☒	☐ ChIP-seq
☒	☐ Flow cytometry
☒	☐ MRI-based neuroimaging
